# Preparation of a Series of Photoresponsive Polymersomes Bearing Photocleavable a 2-nitrobenzyl Group at the Hydrophobic/Hydrophilic Interfaces and Their Payload Releasing Behaviors

**DOI:** 10.3390/polym11081254

**Published:** 2019-07-29

**Authors:** Shota Yamamoto, Takafumi Yamada, Genki Kubo, Kazuo Sakurai, Kazuo Yamaguchi, Jun Nakanishi

**Affiliations:** 1 International Center for Materials Nanoarchitectonics (WPI-MANA), National Institute for Materials Science (NIMS), 1-1 Namiki, Tsukuba, Ibaraki 305-0044, Japan; 2 Department of Chemistry, Kanagawa University, 2946 Tsuchiya, Hiratsuka, Kanagawa 259-1293, Japan; 3 Department of Chemistry and Biochemistry, The University of Kitakyushu, 1-1 Hibikino, Wakamatsu-ku, Kitakyushu, Fukuoka 808-0135, Japan; 4 Graduate School of Advanced Science and Engineering, Waseda University, 3-4-1 Okubo, Shinjuku-ku, Tokyo 169-8555, Japan

**Keywords:** photoresponsive polymersome, photocleavable diblock copolymer, controlled release, self-assembly, drug delivery

## Abstract

In this study, the structure-function relationships of a series of polymersomes composed of well-defined amphiphilic diblock copolymers were investigated. The building blocks were synthesized by clicking hydrophobic polymers, synthesized beforehand, and commercially available poly(ethylene glycol) with photocleavable 2-nitrobenzyl compounds bearing alkyne and maleimide functionalities. All of the tested polymersomes preserved their hollow structures even after sufficient photoirradiation. Nevertheless, the release rate of an entrapped anionic fluorophore was highly dependent on the molecular weight and the type of hydrophobic polymer, as well as on the presence or absence of the charged end groups. Moreover, the polymersomes with a 2-nitrosobenzyl photolysis residue within the hydrophobic shells exhibited photo-induced payload release after complete photolysis. It was concluded that the payload release was mediated by photo-induced permeability changes of the hydrophobic shells rather than the decomposition of their overall structures.

## 1. Introduction

Amphiphilic diblock copolymers self-assemble into diverse nanostructures such as micelles, vesicles, and nanogels, depending on the contents of the hydrophilic/hydrophobic segments. Owing to their structural tunability and the ability to accommodate various compounds, these nanostructures are promising drug carriers [1,2]. In particular, diblock copolymers with a hydrophobic content above 60% form hollow spherical structures with a hydrophobic shell and an aqueous core, which is suitable for encapsulating hydrophilic substances [3,4,5,6]. Even though such polymeric vesicles, called polymersomes, have topologically the same structure as liposomes, the robust thick polymeric membranes make them more stable in biological environments.

Typically, polymersomes are formed from biocompatible diblock copolymers, such as poly(lactic acid)-*b*-poly(ethylene glycol) (PLA-PEG) [7], poly(ε-caprolactone)-*b*-poly(ethylene glycol) (PCL-PEG) [8], and poly(styrene-*b*-acrylic acid) (PS-PAA) [9]. Various biological compounds, including drugs [10], genes [11,12], and nucleic acids [13] are entrapped within their hydrophilic cores. One of the recent trends in this field is the development of stimuli-responsive polymersomes [14,15] because they allow for the spatiotemporally controlled release of entrapped drugs in response to external stimuli such as pH [16], light [17,18], and temperature [19]. Thus, photoresponsive polymersomes have advantages over others in terms of high spatiotemporal resolution and bioorthogonality. One of the most commonly used photoresponsive groups is the 2-nitrobenzyl group, which cleaves upon absorption of near-UV light under ambient conditions.

To date, two kinds of photoresponsive polymersomes based on the 2-nitrobenzyl group have been reported. In the first type, the photoswitch was introduced as the photocleavable side chains of the hydrophobic block of the amphiphilic diblock copolymers. For example, Song and coworkers reported photoresponsive polymersomes based on poly(ethylene glycol)-*block*-poly(*γ*-(4,5-dimethoxy-2-nitrobenzyl)-L-glutamate) (PEG-*b*-PGlu) [20]. The releasing mechanisms of these “*graft*-type” photoresponsive polymersomes are straightforward; photoirradiation produces a hydrophilic carboxylate in the hydrophobic block, which leads to polymersome disassembly by loss of amphiphilicity of the diblock copolymers.

In the second type, the photocleavable group is introduced as a junction of hydrophobic and hydrophilic blocks. The first report for this “*linker*-type” was made by Burdick and coworkers with the PEG-PCL deblock [21], later followed by Meier and coworkers with the hydrophilic PAA and hydrophobic poly(methylcaprolactone) (PMCL) segments [22,23]. Interestingly, these polymersomes reportedly follow different pathways of decomposition as well as photoreleasing processes in response to photoirradiation. Specifically, the former polymersomes retain their hollow structures, which gradually collapse with thickened shells and lead to the expulsion of aqueous contents. On the other hand, the latter polymersomes disassemble into smaller micelles associated with the release of aqueous contents. The authors concluded that the major driving force for the decomposition was the electrostatic repulsion between the photochemically produced anionic carboxy group at the end of the hydrophobic blocks, even though the same anionic group was formed in the PEG-PMCL diblock as well [24].

Such inconsistent fates of the “*linker*-type” photoresponsive polymersomes in the earlier reports can be attributed to the differences in the compositions of block copolymers used in these studies, i.e., (1) the nature of the polymer species constituting the hydrophobic chains (PCL vs. PMCL), (2) hydrophobic/hydrophilic weight ratio of the amphiphilic polymers (0.22 vs. 0.11), (3) type of the photocleavage linker (nitrobenzyl ester vs. nitrobenzyl alanine). Therefore, more systematic studies are needed to clarify the working principle of the “*linker*-type” photoresponsive polymersomes by arbitrarily changing these parameters. However, such studies have been hampered by the poor compatibility of the 2-nitrobenzyl group and the living radical polymerization reactions, which are critical for precise synthesis of the building blocks.

**Figure 1:**
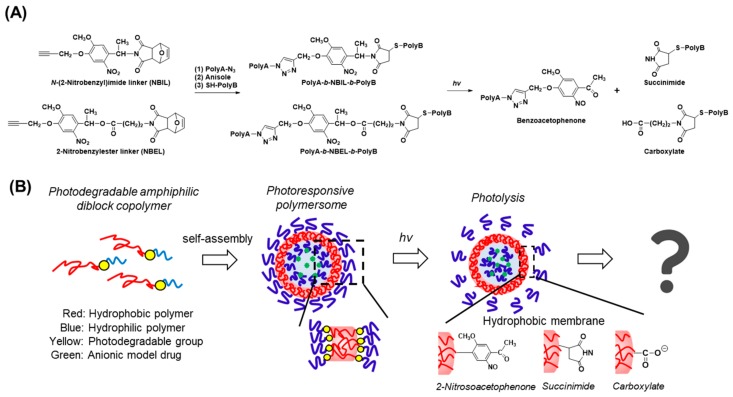
Research strategy of the present study. (**A**) Synthesis and photolysis procedures of photodegradable amphiphilic diblock copolymers based on two different photocleavable heterobifunctional crosslinking linkers (NBIL and NBEL). (**B**) Self-assembly of photocleavable diblock copolymers into “*linker*-type” photoresponsive polymersomes entrapped with a hydrophilic payload. Near-UV irradiation cleaves at the hydrophobic/hydrophilic interfaces yielding various end groups (benzoacetophenone, succinimide, or carboxylate) at the outer and inner interfaces of the polymersomes. The final structures and the payload release mechanisms are as yet unknown.

To overcome this technical barrier, herein we have demonstrated a new research strategy for the analysis of the photorelease mechanism of a series of photoresponsive polymersomes composed of extremely well-defined diblock copolymers (M_w_/M_n_ ≤ 1.07). It is based on the heterobifunctional photocleavable 2-nitrobenzylimide linker molecule (NBIL), as reported previously, which bears alkynyl and maleimide groups at each end (Figure 1A) [25,26]. In addition, we newly synthesized a similar photocleavable 2-nitrobenzyl ester linker molecule (NBEL). These molecules can react with various well-defined hydrophobic/hydrophilic polymers bearing azido or thiol groups, synthesized in advance by living radical polymerization or ring-opening polymerization reactions, via two types of click chemistry, to afford a wide variety of photocleavable diblock copolymers in high yield. Moreover, depending on the arrangement of the hydrophilic/hydrophobic blocks, the residual group formed at the polymersome surface changes from benzoacetophenone to succinimide or anionic carboxylate (Figure 1B). Consequently, we were able to study the impact of the molecular weight, nature of the polymer species of each block, and the photocleavage residues on the structures and photoreleasing properties of the polymersomes.

## 2. Materials and Methods

### 2.1. Materials

All reagents were purchased from Wako Pure Chemical Industries Ltd. (Osaka, Japan), TCI Co., Ltd. (Tokyo, Japan), Sigma-Aldrich (St. Louis, MO, USA), or Kanto Chemical Co., Inc. (Tokyo, Japan), unless otherwise stated. Photodegradable diblock copolymers (PS-*b*-NBIL-*b*-PEG 1a–c, PCL-*b*-NBIL-*b*-PEG 2, and PEG-*b*-NBIL-*b*-PS 3) were synthesized according to the reported methods [25,26].

### 2.2. Synthesis of 1-(4-Methoxy-2-nitro-4-prop-2-ynyloxyphenyl)ethyl Bromide

1-(5-methoxy-2-nitro-4-prop-2-ynyloxyphenyl)ethanol (2.64 g, 16.8 mmol), 1-ethyl-3-(3-dimethylaminopropyl)carbodiimide (EDC.HCl; 3.23 g, 16.8 mmol), 4-dimethyl aminopyridine (DMAP; 1.54 g, 12.6 mmol), 4-bromo-*n*-butyric acid (3.51 g, 21.0 mmol), and dry tetrahydrofuran (THF, 20 mL) were mixed together in a 100 mL flask. The resulting solution was stirred for 15 h at 0 °C under nitrogen. Subsequently, the reaction mixture was concentrated in a rotary evaporator and the residue was extracted with chloroform and water. The combined organic layers were washed with saturated NaCl aqueous solution, dried over magnesium sulfate, filtered, and then concentrated using a rotary evaporator. The obtained residue was purified on a silica gel column using hexane and ethyl acetate (2:1) to afford 1-(4-methoxy-2-nitro-4-prop-2-ynyloxyphenyl)ethyl bromide as a yellow solid in 69% yield (2.90 g, 7.25 mmol). ^1^H NMR (400 MHz, CDCl_3_): δ7.76 (1H, s), 7.04 (1H, s), 6.51 (1H, q, *J* = 6.4 Hz), 4.82 (2H, d, *J* = 2.3 Hz), 3.99 (3H, s), 3.44 (2H, m), 2.57 (2H, m), 2.17 (2H, m), and 1.63 (3H, d, *J* = 6.4 Hz).

### 2.3. Synthesis of the Heterobifunctional Linker NBEL

7-oxabicyclo[2.2.1]hept-5-ene-dicarboxyimide (an adduct of maleimide with furan, 1.20 g, 7.27 mmol), K_2_CO_3_ (5.01 g, 36.3 mmol), and dry DMF (40 mL) were added to a 300 mL two-necked flask. The mixture was stirred for 30 min at room temperature under nitrogen. Next, 1-(4-methoxy-2-nitro-4-prop-2-ynyloxyphenyl)ethyl bromide (2.90 g, 7.25 mmol) was added to the flask and the resulting solution was stirred for 3 h at room temperature under nitrogen. Subsequently, the reaction mixture was concentrated in a rotary evaporator and the residue was extracted with chloroform and water. The combined organic layers were washed with saturated NaCl aqueous solution, dried over magnesium sulfate, filtered, and concentrated in a rotary evaporator. The residue was purified on a silica gel column using chloroform and methanol (9:1) to afford the heterobifunctional linker as a yellow solid in 83% yield (2.93 g, 6.05 mmol). ^1^H NMR (400 MHz, CDCl_3_): δ7.74 (1H, s), 7.04 (1H, s), 6.51 (1H, m), 6.47 (1H, q, J = 6.4 Hz), 5.23 (2H, m), 4.82 (2H, d, *J* = 2.4 Hz), 3.99 (3H, s), 3.44 (2H, m), 2.83 (1H, d, *J* = 6.4 Hz), 2.74 (1H, d, *J* = 6.4 Hz), 2.58 (1H, t, *J* = 2.4 Hz), 2.35 (2H, m), 1.90 (2H, m), and 1.62 (3H, d, *J* = 6.4 Hz).

### 2.4. Synthesis of 4-Methyl-ε-caprolactone (MCL)

In a 1 L flask, *m*-chloroperbenzoic acid (28.6 g, 166 mmol), 4-methylcyclohexanone (9.96 g, 88.8 mmol), and dichloromethane (600 mL) were mixed together. The resulting solution was stirred for 24 h at room temperature. The reaction flask was immersed in a cold bath (at −78 °C) and the generated by-products were removed by filtration. The mixture was concentrated in a rotary evaporator and the residue was extracted with chloroform and water. The combined organic layers were washed with saturated NaCl aqueous solution, dried over magnesium sulfate, filtered, and then concentrated in a rotary evaporator. The residue was evacuated with a high-vacuum oil pump to completely remove the organic solvent. Finally, MCL was obtained as a yellow oil (9.35 g). ^1^H NMR (400 MHz, CDCl_3_): δ4.28 (1H, m), 2.61 (3H, m), 1.52 (5H, m), and 0.99 (3H, m).

### 2.5. Synthesis of Poly(4-methyl-ε-caprolactone) (PMCL)

In a 20-mL two-necked flask, H_2_O (1.3 μL, 0.0690 mmol), MCL (0.973 g, 7.59 mmol), and dry toluene (7.5 mL) were mixed and freeze-dried three times. Next, diphenyl phosphate (0.0360 g, 0.144 mmol) was added to the flask and the resulting solution was stirred for 52 h at 0 °C under nitrogen. Subsequently, the vessel was evacuated with a high-vacuum oil pump to completely remove toluene from the product. The obtained residue was then dissolved in dichloromethane (1.0 mL) and precipitated by adding 100 mL of cold hexane. The precipitates were recovered by centrifugation (4000 rpm, 10 min, thrice) to afford PMCL as a clear paste (0.187 g, M_n_ = 4200). The PMCL product thus obtained was evacuated with a high-vacuum oil pump to completely remove water and subsequently used for the next post polymerization reaction.

The above PMCL (0.177 g, 0.0421 mmol, M_n_ = 4200), MCL (0.540 g, 4.21 mmol), and dry toluene (4.0 mL) were added to a 20 mL two-necked flask and the mixture was freeze-dried three times. Next, diphenyl phosphate (0.0310 g, 0.126 mmol) was added to the flask and the resulting solution was stirred for 7 h at 0 °C under nitrogen. Subsequently, the reaction mixture was precipitated from 100 mL of cold methanol. The precipitates were recovered using a centrifuge (4000 rpm, 10 min, thrice) to afford PMCL as a clear paste in 64% yield (0.182 g, 0.0268 mmol, M_n_ = 6,800). ^1^H NMR (400 MHz, CDCl_3_): δ4.10 (106H, m), 3.77 (2H, m), 2.30 (106H, m), 1.60 (302H, m), and 0.92 (160H, m)

### 2.6. Synthesis of α-Carboxyl-ω-tert-butyldimethylsilyl Group-Terminated Poly(α-methyl-ε-caprolactone) (HOOC-PMCL-OTBDMS)

PMCL (0.111 g, 0.0163 mmol, M_n_ = 6,800), *tert*-butyldimethylchlorosilane (TBDMS-Cl; 0.0491 g, 0.326 mmol), imidazole (0.0270 g, 0.397 mmol), and dry DMF (0.3 mL) were placed in a 20 mL two-necked flask. The resulting solution was stirred for 17 h at room temperature under nitrogen. Subsequently, DMF (370 μL) and water (70 μL) were added to the flask and the reaction mixture was precipitated from 20 mL of cold hexane. The precipitates were recovered by centrifugation (4000 rpm, 10 min, thrice) and HOOC-PMCL-OTBDMS was ultimately obtained as a clear paste in 98% yield (0.110 g, 0.0159 mmol, M_n_ = 6,800). ^1^H NMR (400 MHz, CDCl_3_): δ4.10 (106H, m), 3.70 (2H, m), 2.30 (106H, m), 1.53 (370, m), 0.92 (166H, m), and 0.041 (6H, s).

### 2.7. Synthesis of α-2-(2′,4′-Dinitrophenylthio)ethyl-ω-tert-butyldimethylsilyl-terminated Poly(α-methyl-ε-caprolactone) (DNP-PMCL-TBDMS)

HOOC-PMCL-OTBDMS (0.110 g, 0.0159 mmol), 2-(2′,4′-dinitrophenylthio)ethanol (0.389 g, 1.59 mmol), 4-dimethylaminopyridine (DMAP) (0.194 g, 1.59 mmol), 1-(3-dimethylaminopropyl)-3-ethylcarbodiimide hydrochloride (EDC.HCl; 0.306 g, 1.59 mmol), and dry chloroform (1.2 mL) were added to a 20 mL two-necked flask. The resulting solution was stirred for 84 h at room temperature under nitrogen. Subsequently, the reaction mixture was precipitated from 20 mL of cold hexane. The precipitates were recovered by centrifugation (4000 rpm, 10 min, thrice) to afford DNP-PMCL-TBDMS as a yellow paste in 89% yield (0.096 g, 0.0141 mmol, M_n_ = 6,900). ^1^H NMR (400 MHz, CDCl_3_): δ9.09 (0.9H, d, *J* = 2.5 Hz), 8.43 (0.9H, dd, *J* = 9.0 and 2.5 Hz), 7.78 (0.9H, d, *J* = 9.0 Hz), 4.37 (1.8H, t, *J* = 6.9 Hz), 4.10 (106H, m), 3.70 (2H, m), 3.34 (1.8H, t, *J* = 6.9 Hz), 2.30 (106H, m), 1.40 (372H, m), 0.92 (160H, m), and 0.041 (6H, s).

### 2.8. Synthesis of α-Thiol-ω-tert-butyldimethylsilyl-terminated Poly(α-methyl-ε-caprolactone) (HS-PMCL-TBDMS)

In a 20-mL two-necked flask, DNP-PMCL-TBDMS (0.0950 g, 0.0133 mmol), 2-mercaptoethanol (1.03 g, 13.3 mmol), and triethylamine (five drops) were mixed together. The resulting solution was stirred for 18 h at room temperature under nitrogen. The reaction mixture was subsequently precipitated from 20 mL of cold hexane. The precipitates were recovered using a centrifuge (4000 rpm, 10 min, thrice) to afford HS-PMCL-OTBDMS as a clear paste in 82% yield (0.0720 g, 0.0109 mmol, M_n_ = 6,600). ^1^H NMR (400 MHz, CDCl_3_): δ4.10 (103H, m), 3.70 (2H, m), 3.34 (2H, t, *J* = 6.9 Hz), 2.30 (107H, m), 1.50 (428H, m), 0.92 (154H, m), and 0.041 (6H, s).

### 2.9. Synthesis of PEG_750_-b-NBEL

NBEL (0.204 g, 0.421 mmol), azido-terminated PEG (M_n_ = 750; 0.405 g, 0.529 mmol), CuBr (0.0600 g, 0.418 mmol), *N,N,N′,N″,N″*-pentamethyldiethylenetriamine (PMDETA; 86 μL, 0.421 mmol), and dry DMF (10 mL) were mixed together in a 30 mL two-necked flask. The resulting solution was stirred for 18 h at room temperature under nitrogen, and then the vessel was evacuated with a high-vacuum oil pump to completely remove DMF. Subsequently, the residue was extracted with chloroform and water. The combined organic layers were washed with 0.065 M ethylenediaminetetraacetic acid (EDTA) aqueous solution, dried over magnesium sulfate, filtered, and then concentrated in a rotary evaporator. The residue was purified on a silica gel column using chloroform and methanol (9:1) to afford PEG_750_-*b*-NBEL in a yellow paste form in 82% yield (0.433 g, 0.347 mmol). ^1^H NMR (400 MHz, CDCl_3_): δ7.89 (1H, s), 7.77 (1H, s), 7.02 (1H, s), 6.51 (2H, s), 6.45 (1H, d, *J* = 6.4 Hz), 5.29 (2H, s), 5.23 (2H, d, *J* = 7.8 Hz), 4.56 (2H, t, *J* = 5.0 Hz), 3.96 (3H, s), 3.88 (2H, t, *J* = 5.0 Hz), 3.65 (85H, m), 3.38 (3H, s), 2.83 (1H, d, *J* = 6.9 Hz), 2.75 (1H, d, *J* = 6.4 Hz), 2.35 (2H, m), 1.89 (2H, m), and 1.61 (3H, d, *J* = 6.9 Hz).

### 2.10. Synthesis of PEG_750_-b-NBEL-Maleimide by Furan Deprotection

PEG_750_-*b*-NBEL (0.433 g, 0.347 mmol) and anisole (4.0 mL) were added to a 30 mL two-necked flask. The resulting solution was stirred for 4 h at 120 °C under nitrogen. Subsequently, the vessel was evacuated with a high-vacuum oil pump to completely remove anisole. The residue was purified on a silica gel column using chloroform and methanol (9:1) to afford PEG_750_-*b*-NBEL-maleimide as a yellow paste in 95% yield (0.386 g, 0.267 mmol). ^1^H NMR (400 MHz, CDCl_3_): δ7.89 (1H, s), 7.77 (1H, s), 7.03 (1H, s), 6.66 (2H, s), 6.46 (1H, q, *J* = 6.5 Hz), 5.30 (2H, s), 4.56 (2H, t, *J* = 5.2 Hz), 3.97 (3H, s), 3.88 (2H, t, *J* = 5.2 Hz), 3.65 (85H, m), 3.38 (3H, s), 2.36 (2H, m), 1.92 (2H, m), and 1.62 (3H, m).

### 2.11. Synthesis of PEG_750_-b-NBEL-b-PMCL_6600_ 5

PEG_750_-*b*-NBEL-maleimide (0.0104 g, 0.00862 mmol), HS-PMCL-TBDMS (0.0700 g, 0.00920 mmol), and dry THF (1.0 mL) were added to a 10 mL two-necked flask. The resulting solution was stirred for 20 h at room temperature under nitrogen. Subsequently, the product was precipitated from 50 mL of cold hexane. The precipitates were recovered using a centrifuge (4000 rpm, 2 × 10 min). The residue was purified on a silica gel column using chloroform and methanol (9:1) to afford *PEG_750_-b-NBEL-b-PMCL_6600_* 5 as a yellow paste in 58% yield (0.0400 g, 0.00498 mmol). ^1^H NMR (400 MHz, CDCl_3_): δ7.88 (1H, s), 7.76 (1H, s), 7.00 (1H, s), 6.43 (1H, q, *J* = 6.5 Hz), 5.29 (2H, s), 4.54 (2H, m), 4.09 (103H, m), 3.95 (3H, s), 3.85 (3H, m), 3.61 (75H, m), 3.37 (3H, s), 3.23 (1H, m), 3.01 (2H, m), 2.30 (107H, m), 1.55 (280H, m), 0.90 (154H, m), and 0.030 (6H, m).

### 2.12. Preparation of Photoresponsive Polymersomes

Diblock copolymers (**1a**–**c**, **2**–**5**) were first dissolved in THF and then added drop-wise to 10 mM phosphate buffer (pH = 7.4). The concentration of the polymer in this solution was 0.8 mg mL^−1^. After stirring for 2 h, THF was removed by dialysis against fresh 10 mM phosphate buffer (pH = 7.4) for a day using a 3.5 kD molecular weight cut-off (MWCO) dialysis membrane.

### 2.13. Preparation of Photoresponsive Polymersomes Encapsulating Fluorescein

The polymer solutions were added drop-wise to solution of 100 mM fluorescein (self-quenching concentration) in 10 mM phosphate buffer (pH = 7.4). The concentration of the polymer in this solution was 0.8 mg mL^−1^. After stirring for 2 h, non-encapsulating fluorescein was removed by dialysis against fresh 10 mM phosphate buffer (pH = 7.4) for three days in the dark using a 20 kD MWCO dialysis membrane.

### 2.14. Characterization of Photoresponsive Polymersomes

The diameters and particle size distributions of the polymersomes were determined by dynamic light scattering (DLS, Beckman Coulter, Brea, CA, USA). The prepared polymersome solutions were placed in a disposable sizing cuvette. The measurements were performed at a scattering angle of 90° at room temperature.

The hydrodynamic radius (*R_h_*) and radius of gyration (*R_g_*) of the polymersomes were measured by field flow fractionation coupled with light scattering. An Eclipse asymmetric-flow-field-flow-fractionation (AF4) separation system was connected with DAWN HELEOS II 8 multiangle light scattering (LS) detector (Wyatt Technology, Dernbach, Germany). A flat channel has a length of 17.4 cm and spacer height of 350 μm. The polyethersulfone (PES) membrane (5kDa) was attached to the bottom of the Wyatt channel. An Agilent 1200 Infinity Series Infinitely Better Isocratic Pump (Agilent technologies, Waldbronn, Germany) was used to control the carrier flow and sample injection. Before starting the experiments, 10 mM phosphate buffer (pH = 7.4) was circulated in a separation system. Polymersome solution (0.8 mg mL^−1^) was supplied in a Wyatt channel. The separation program was as follows: Step 1: Elution with cross-flow at 1 min (detector-flow 1.0 mL min^−1^, and cross-flow 0.6 mL min^−1^), Step 2: Focus-flow at 1 min (focus-flow 0.6 mL min^−1^), Step 3: Injection with focus-flow at 5 min (detector-flow 1.0 mL min^−1^, focus-flow 0.6 mL min^−1^, and inject-flow 0.2 mL min^−1^), Step 4: Focus-flow at 4 min (detector-flow 1.0 mL min^−1^, and focus-flow 0.6 mL min^−1^), Step 5: Elution with cross-flow at 25 min (detector-flow 1.0 mL min^−1^, and cross-flow 0.6–0.1 mL min^−1^), Step 6: Elution with cross-flow at 5 min (detector-flow 1.0 mL min^−1^, and cross-flow 0.1 mL min^−1^), Step 7: Elution without cross-flow at 3 min (detector-flow 1.0 mL min^−1^, and inject-flow 0.2 mL min^−1^). The ASTRA software (Wyatt Technology, Dernbach, Germany) was used to obtain geometric diameters on the basis of the angular dependence of scattered light recorded by the multi angle light scattering (MALS) detector.

The hydrophobic thicknesses and structures of the polymersomes were determined by small angle X-ray scattering (SAXS) measurements at BL-40B2 of SPring-8, Japan. Polymersome solutions were placed into a quartz capillary cell (Hilgenberg GmbH, Malsfeld, Germany) with a light path of 2 mm. The incident X-ray wavelength (λ) was fixed at 0.071 nm and the scattering was measured with sample-to-detector distances of 1 or 4 m, combining the two profiles afterward to obtain *I*(q) versus *q* plots, where *I*(q) and *q* refer to the excess scattering intensity and the absolute value of the scattering vector, respectively. These sample-to-detector distances and wavelength provided a range of 0.01 to 10 nm^−1^ for *q*.

The structures of the polymersomes in the vacuum state were observed by transmission electron microscopy (TEM). A drop of the polymersome solution was placed on a 200-mesh copper grid treated with glow discharge to make it hydrophilic. After 5 min, the polymersome solution was removed from the grid and the polymersome was negatively stained by phosphotungstic acid solution for 15 min. Then, the grid was rinsed with 10 mM phosphate buffer to remove the excess stain solution. TEM micrographs were acquired using the JEOL JEM 2100F TEM instrument (Tokyo, Japan) operated at 200 kV.

### 2.15. Photoirradiation

The polymersome solution was placed in a quartz cell and irradiated with near-UV light from an Hg arc lamp (USHIO, BA-H250, Tokyo, Japan) in an Optical Modulex (USHIO, H250) through copper sulfate solution (λ > 320 nm). Samples were irradiated with an intensity of 25 mW cm^−2^, for a given amount of time at room temperature.

### 2.16. Release of Fluorescein from the Polymersome

The polymersome encapsulating fluorescein solution was placed in a quartz cell and then the fluorescence intensity (F_0_) at 520 nm was measured by a UV-Vis spectrophotometer (JASCO, V-570, Tokyo, Japan). The polymersome solutions were irradiated as the above method. The value of fluorescence intensity (F_t_) was measured for a given time. After the addition of 10% Triton X-100, fluorescence intensity of the sample (F_100_) was measured. The release rate of fluorescein from the polymersome was calculated using the formula: [(F_t_ − F_0_)/(F_100_ − F_0_)] × 100.

## 3. Results and Discussion

### 3.1. Design of Diblock Copolymers and Characterization

In order to directly compare the impact of the molecular weight, hydrophobic polymer species, and the photocleaved residual groups on the structures as well as the photoresponse characteristics of the polymersomes, we started from a common photocleavable heterobifunctional *N*-(2-nitrobenzyl)imide linker (NBIL) (Figure 1: Left), which was developed in our previous studies [25,26]. This molecule allowed us to conjugate well-defined hydrophobic and hydrophilic polymers, synthesized beforehand, via two kinds of click chemistry, namely, Huisgen cycloaddition and Michael addition, at the end alkynyl and maleimide group, respectively (Figure 1A: Middle). Furthermore, the diblocks could be cleaved at the hydrophobic/hydrophilic interfaces upon subsequent photoirradiation (Figure 1A: Right). In addition, we newly synthesized another heterobifunctional photocleavable linker, based on 2-nitrobenzylester (NBEL, Figure 1A: Left). This molecule underwent the same two kinds of click chemistry but, because of the use of an ester linkage instead of an imide (Figure 1A: Middle), it produced an anionic carboxy group after photocleavage (Figure 1A: Right). NBEL was synthesized by following the procedure shown in Figure S1. Briefly, compound 8 was synthesized as reported. Compound 9 was synthesized in 63% yield from 8 and 4-bromo-*n*-butyric acid by dehydration condensation. Finally, the heterobifunctional linker 2 was obtained in 62% yield by Williamson synthesis from 9 and 7-oxabicyclo[2.2.1]hept-5-ene-dicarboxyimide. The new compounds were confirmed by ^1^H NMR.

**Figure 2:**
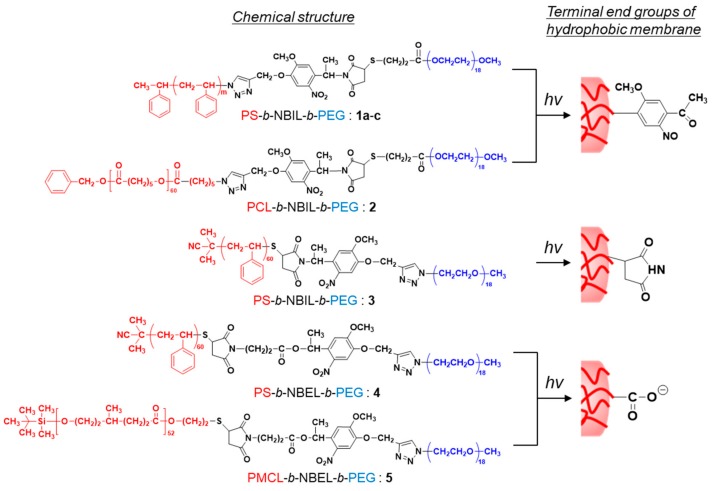
Chemical structures of photocleavable amphiphilic diblock copolymers used in this study. The hydrophobic and hydrophilic segments are shown in red and blue, respectively. The terminal end groups produced at the inner and outer interfaces of the polymersomes after photolysis are shown on the right.

Figure 2 shows the chemical structures of the photocleavable diblock copolymers used in this study. **1a–c**, **2**, and **3** are the diblock copolymers prepared from NBIL. **1a–c** and **3** are PS-*b*-NBIL-*b*-PEG, where the configurations of the PEG/PS blocks are opposite. Therefore, 2-nitoroso-acetophenone and succinimide were left behind at the end of the hydrophobic segments for **1a–c** and **3**, respectively. Among the PS-*b*-NBIL-*b*-PEG copolymers, the molecular weight of the PS segments was varied as 4000 (**1a**), 6000 (**1b** and **3**), and 8000 (**1c**). On the other hand, **2** was PCL-*b*-NBIL-*b*-PEG. Although it was similar to **1b**, PCL was used instead of PS as the hydrophobic block. **4** (PS-*b*-NBEL-*b*-PEG) and **5** (PMCL-*b*-NBEL-*b*-PEG) were the diblock copolymers prepared from NBEL (Figure S1). After the photocleavage of these block copolymers, the anionic carboxyl group was produced at one end of the hydrophobic block. In this manner, it was possible to study the effect of the negatively charged group, which has been reported to be essential in previous work [22,23], on the decomposition of polymersomes. By choosing appropriate reaction conditions of atomic transfer radical polymerization (ATRP) and reversible addition-fragmentation chain transfer (RAFT) polymerization for PS and ring-opening polymerization for PCL and PMCL, **1b**/**3**/**4 2**, and **5** were synthesized such that they had similar hydrophobic chain lengths, but with different hydrophobic polymers, i.e., PS, PCL, and PMCL. For all of the synthesized diblock polymers, the molecular weight of the hydrophilic segment was kept constant by using commercial PEG (M_w_ = 750). Table 1 shows the molecular characterization data of the photocleavable diblock copolymers. All of the diblock copolymers had narrow molecular distributions (Figure S2, M_w_/M_n_ < 1.08). The hydrophilic PEG contents in the polymers varied from 8.0 to 13.2 wt.%, which was within a suitable range for polymersome forming diblock copolymers [27].

### 3.2. Structural Characterization of Photoresponsive Polymersomes

**Figure 3:**
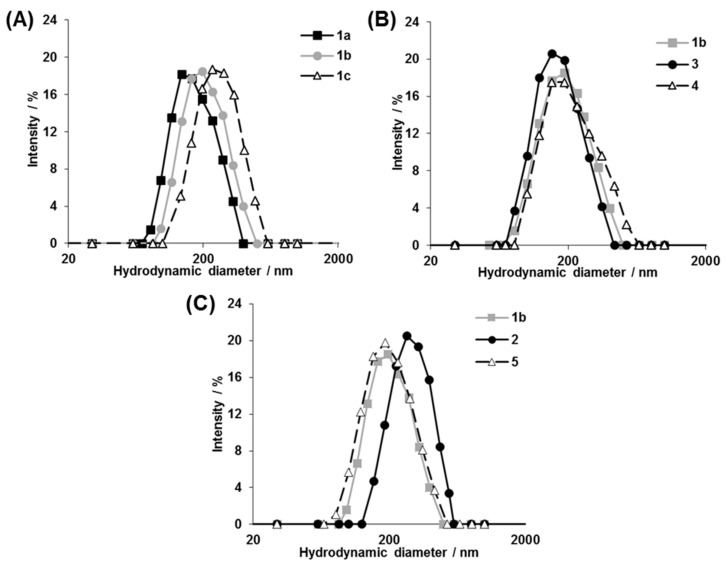
Dynamic light scattering (DLS) characterization of photoresponsive polymersomes. (**A**) PS-based polymersomes with varied molecular weight at the hydrophobic segments: **1a**, 4000; **1b**, 6000; and **1c**, 8000. (**B**) PS-based polymersomes, which produced different end groups after photolysis: **1b**, nitrosoacetophenone; **3**, succinimide; and **4**, carboxylate. (**C**) Polymersomes bearing different hydrophobic polymer species: **1b**, PS; **2**, PCL; and **5**, PMCL. For b and c, the molecular weight of the hydrophobic blocks is almost identical (6000). The DLS measurements were conducted for as-prepared polymersomes.

From these diblock copolymers, a series of photoresponsive polymersomes were prepared by the cosolvent method [28]. DLS measurements demonstrated the formation of monodisperse nanostructures, ranging from 155 to 240 nm, with polydispersity (PDI) being on average 0.160 (Figure 3). Among **1a–c**, the diameter of the polymersomes became larger as the molecular weight of the hydrophobic PS segments increased (Figure 3A). The results were consistent with a previous report [27], where various non-photoresponsive polymersomes were prepared from diblock copolymers without photocleavable groups. It is noteworthy that regardless of the arrangements of hydrophilic/hydrophobic blocks and the type of the photocleavage linker, the diameter of PS-based polymersomes **1b**, **3**, and **4** was almost identical as long as the molecular weight of the PS segment was the same (Figure 3B). On the other hand, considering the polymersomes made from similar molecular weight diblock copolymers with different hydrophobic polymer species of PS, PCL, and PMCL for **1b**, **2**, and **5**, respectively, those bearing PCL had larger diameters than those bearing PS and PMCL (Figure 3C, 240 for 2 vs. 190 and 180 nm for **1b** and **5**, respectively). It should be emphasized that such systematic analysis of the relationship between the polymersome size and the polymer composition became possible only because we were able to obtain various photoresponsive diblock copolymers via the synthetic strategy reported here.

**Figure 4:**
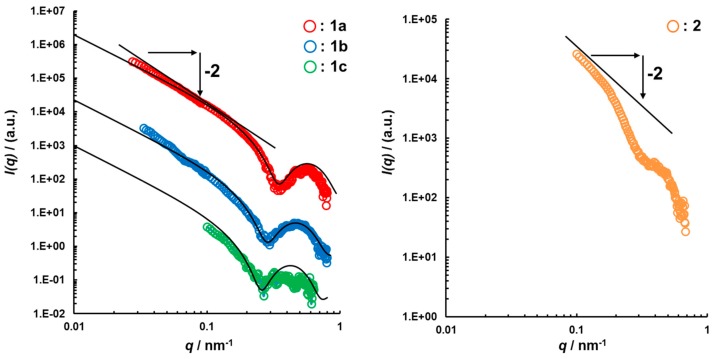
Small angle X-ray scattering (SAXS) profile and structural parameters of polymersomes (**A**) **1a**–**c** and (**B**) **2**.

To confirm the formation of the characteristic hollow spherical structures of the polymersomes, we analyzed them by SAXS. The profile and the structural parameters are shown in Figure 4. For all analyzed samples, the SAXS profiles agreed with the model fitting of the vesicle and the scattering vector was q^−2^, which indicated a vesicle structure [29]. The core radius for half layer thickness of the hydrophobic shell of the polymersomes **1a**–**c** and **2** was 3.3, 4.3, 5.2, and 9.5 nm, respectively. In PS-based polymersomes, the hydrophobic shell thickness increased depending on the extension of the hydrophobic chain length. On the other hand, when the shell thickness was compared for the polymersomes bearing different hydrophobic blocks, i.e., PS and PCL, the half layer thickness of the PCL-based polymersome 2 was found to be twice as long as that of the PS-based polymersomes 1b. These results are reasonable considering the closer solubility parameter of PCL to water than that of PS. Moreover, the thickness of the PEG chains calculated from the difference between the hydrophobic shell and the total shell thickness was almost identical, which was consistent with the same molecular weight of PEG in the block copolymers.

**Figure 5:**
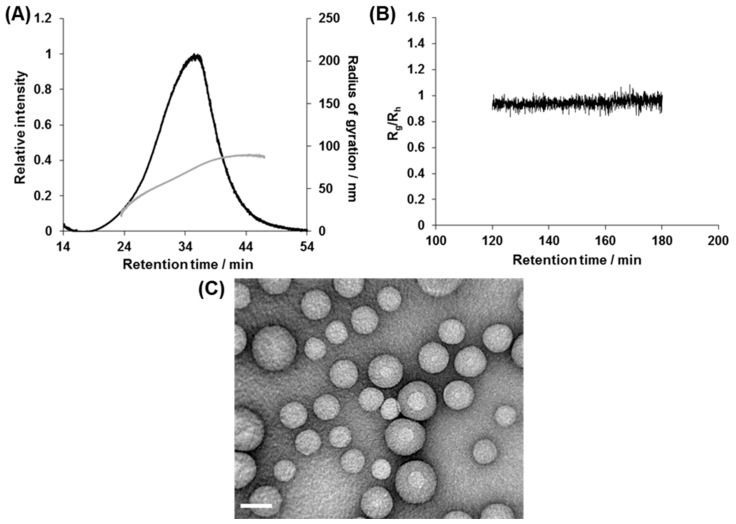
Structural characterization of as-prepared polymersome **1b**. (**A**) AF4 fractogram, (**B**) Rg/Rh plot of **1b**, and (**C**) TEM image. Scale bar: 100 nm.

The polymersome solutions were further characterized by using the AF4-MALS technique, since polymersomes are intrinsically polydisperse. In the AF4 measurements, the samples were separated in a laminar flow with a parabolic flow velocity profile under applied perpendicular (asymmetric) cross flow. This separation method is suitable for the analysis of polymeric assemblies, nanoparticles, and proteins because there is no stationary phase, and consequently there are no interactions that can disassemble, clog, or denature the nanomaterials [30,31,32,33]. Moreover, the online MALS detector allowed us to obtain radius of gyration (*R_g_*) and hydrodynamic radius (*R_h_*) of the fractionated samples by simultaneous measurements of static light scattering and quasi-elastic light scattering (QELS). Figure 5A shows an example of the AF4 fractogram of the polymersomes made from diblock copolymers **1b**. The fractograms showed a single elution peak, indicating a monodisperse distribution. Similar results were obtained with the polymersomes 2, 3, and 5 bearing different hydrophobic groups and having different molecular weight as well (Figure S3). To estimate the morphology of the nanoparticles, the time profile of the shape factor (*R*_g_/*R*_h_) was analyzed based on MALS and QELS data. It is known that the shape factor reflects the morphologies of the nanoparticles, i.e., whether they are spheres, hollows, or rods [34]. Since online QELS measurements can give a wrong estimation of the hydrodynamic radii of nanoparticles because of the modulation of Brownian motion by the detector flow, we conducted the AF4-MALS measurements at an extremely low flow rate (0.2 mL min^−1^) only for this experiment (Figure S4). The *R*_g_/*R*_h_ value between 120 and 180 min of polymersome **1b** was found to be constant at ~1.0, demonstrating the formation of hollow polymersome structures (Figure 5B). The *R*_g_/*R*_h_ value of polymersome **2** was also constant at ~1.0 and similar results were obtained for the polymersomes made from **3** and **5** (Figure S5).

To further confirm the hollow structure, polymersome **1b** was analyzed by TEM. The TEM micrographs revealed the presence of spherical structures of diameters ranging from 50 to 150 nm (Figure 5C). The diameter of the nanoparticles was found to be smaller than that determined from DLS and AF4 analysis, which was presumably because TEM analyzes the dried state of the polymersomes.

### 3.3. Kinetic analysis of the Photolysis Reaction of 2-nitrobenzyl Groups and Photorelease of Entrapped Payload in the Polymersomes

Next, the photoresponsivity of the polymersomes was examined. First, the photolysis reaction of the *N*-(2-nitrobenzyl)imide group in the polymersome solution was analyzed by ^1^H NMR. As an example, the polymersome **1b** solutions were irradiated for a given time period and then the solution was condensed to dissolve the residual photoproducts by CDCl_3_. The obtained spectra showed complete disappearance of peaks at 6.0 and 7.6 ppm, and a new signal appeared at 8.5 ppm after irradiation for 2 min (Figure S6). These results indicated that the rate of the photolysis reaction of *N*-(2-nitrobenzyl)imide was not different from the polymer solution in THF [26]. In a similar fashion, the photolysis reaction of the 2-nitrobenzyl ester-type linker was examined for polymersome **4**, which also showed a comparable photocleavage rate to that observed in its THF solution as well as to that of the 2-nitrobenzylimide-type polymersome **1b** described above (Figure S7).

**Figure 6:**
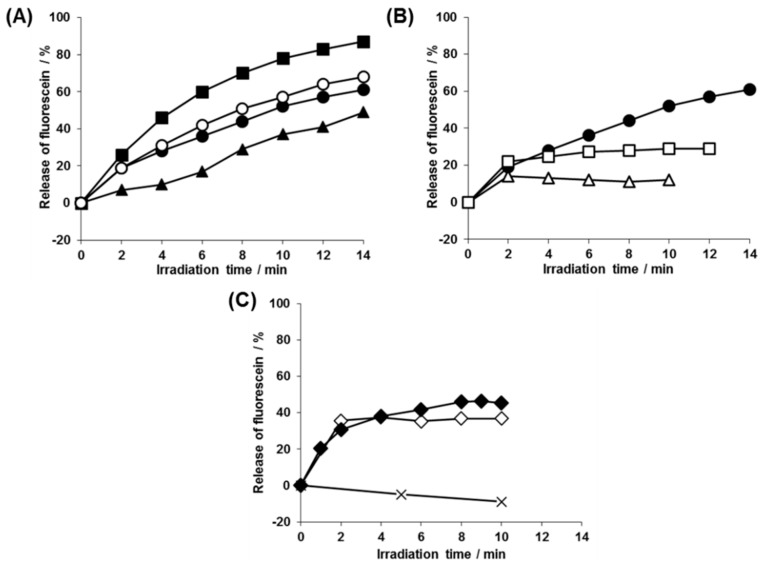
Photorelease behaviors of entrapped fluorescein molecules from the polymersomes evaluated by fluorescence dequenching. (**A**) Impact of molecular weight and polymer species of the hydrophobic segments: PS-based polymersomes **1a** (■), **1b** (●), **1c** (▲), and PCL-based polymersomes **2** (○). (**B**) Impact of prolonged irradiation and the photolysis residual groups: Polymersome **1b** (●, □), with the 2-nitosoacetophenone residue. Polymersome **3**, with the succinimide residue (∆). (**C**) Impact of prolonged irradiation and hydrophobic polymer species: PS-based polymersomes **4** (×) and PMCL-based polymersome **5** (♦, ◊). For plots (□, ◊), polymersomes were initially irradiated for 2 min and then the solutions were kept in the dark. For the other plots, the polymersome solutions were irradiated every 1 min.

To analyze the payload photorelease, we prepared polymersomes **1a**–**c**, **2**, **3**, **4**, and **5** in a self-quenching concentration (100 mM) of fluorescein solution and analyzed their de-quenching process upon release from the polymersome interior to the bulk solution by fluorescence spectroscopy. The polymersomes encapsulating fluorescein showed no fluorescence increase under the dark condition, indicating that they did not leak the payloads without photoirradiation (Table S1). When we analyzed the effect of the molecular weight of the hydrophobic PS block with the polymersomes **1a**–**c**, the photorelease rate became faster with the decreasing molecular weight of the hydrophobic blocks and ~60–80% release of the total entrapped contents was achieved (Figure 6A, ■, ●, ▲). On the other hand, the impact of the polymer species of the hydrophobic block was examined by comparing polymersomes **1b** and **2** as they had different hydrophobic polymer species (PS vs. PCL), but similar molecular weight hydrophobic segments. The photorelease rates of the two polymersomes were almost the same, despite their different hydrophobic shell thicknesses (determined from the SAXS analysis, *vide supra*). Interestingly, these polymersomes (**1a**–**c** and **2**) continued releasing fluorescein even after the completion of the photolysis reaction at 2 min (Figure 6A, ● and ○). The prolonged payload release was not spontaneous leakage because the photorelease remained halted when irradiation was stopped after 2 min and the solution was put in the dark (Figure 6B, □). In contrast, for polymersome 3, where PS and PEG segments were swapped, fluorescein release stopped at 2 min with 20% release of the total contents and no further release was detected by prolonged irradiation (Figure 6B, ∆). To investigate the effect of charged end groups on the photoreleasing behaviors, we examined PS-based polymersomes 4, which formed a carboxy group after photolysis. Interestingly, there was absolutely no photorelease from the beginning (Figure 6C, ×). When we further derived this polymersome to 5, which had the PMCL hydrophobic block instead of PS, it again showed payload photorelease. However, the release significantly slowed down, but not halted, at the time of the completion of the photolysis reaction (2 min; Figure 6C, ♦). In contrast, the release was completely terminated when photoirradiation was stopped at 2 min and the solution was put in the dark (Figure 6B, ◊). These complex characteristics of the payload photorelease from the polymersome solutions raise fundamental questions about the structures of the polymersomes after photoirradiation.

### 3.4. Structural Analysis of the Photoresponsive Polymersomes after Photoirradiation

**Figure 7:**
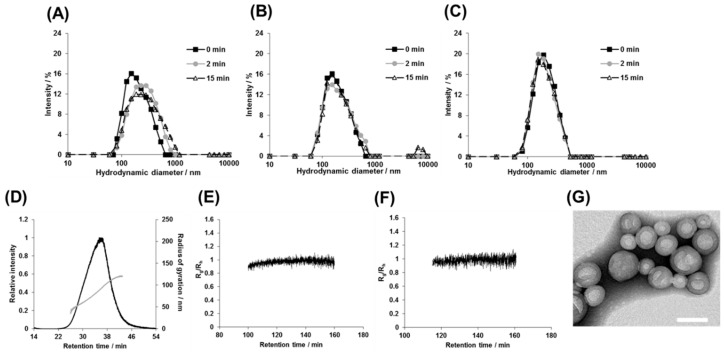
Structural changes in the polymersomes in response to photoirradiation. (**A**–**C**) DLS results of polymersomes (**A**) **1b**, (**B**) **2**, and (**C**) **5**, irradiated for 0 (■), 2 (gray, ♦) and 15 min (∆). (**D**) An AF4 fractogram of polymersome **2** after irradiation for 15 min. (**E**,**F**) Time profiles of the shape factor (Rg/Rh), determined from AF4 fractograms, of polymersomes (**E**) **2** and (**F**) **5** after irradiation for 15 min. (**G**) TEM image of polymersome 1b after irradiation for 15 min. Scale bar = 100 nm.

Therefore, the polymersomes were structurally characterized after photoirradiation by DLS, AF4, and TEM. The batch-type DLS measurement showed slight increase in average diameter (by 20 nm) for the PS-based **1b** and **3** upon irradiation for 2 min. In contrast, there were no significant changes for the other polymersomes (Figure 7A–C and Figure S8, ■ and ♦). The diameter of polymersomes did not change upon prolonged irradiation up to 15 min (Figure 7A–C, ∆) and even much longer (data not shown). AF4 measurements of polymersomes **2** and **5** also showed a similar single peak to that observed before irradiation (Figure 7D and Figure S9). Moreover, the *R*_g_/*R*_h_ value was close to 1.0, which indicated that the hollow structure was maintained (Figure 7E,F). Unfortunately, polymersomes **1a**–**c**, and **3**–**4** did not elute from AF4, presumably because they were aggregated during the focusing (concentrating) step of the AF4 elution process. This aggregation might arise from the highly hydrophobic nature of PS-based polymersomes after photoirradiation. Instead, we obtained TEM micrographs of polymersome 1b, showing a hollow structure even after photoirradiation (Figure 7G). Altogether, these results indicated that all of the polymersomes retained their spherical hollow structures even after irradiation of a sufficient dose of near-UV light for the photolysis of the 2-nitrobenzyl groups.

### 3.5. Expected Mechanism of Fluorescein Photorelease from Polymersomes

**Figure 8:**
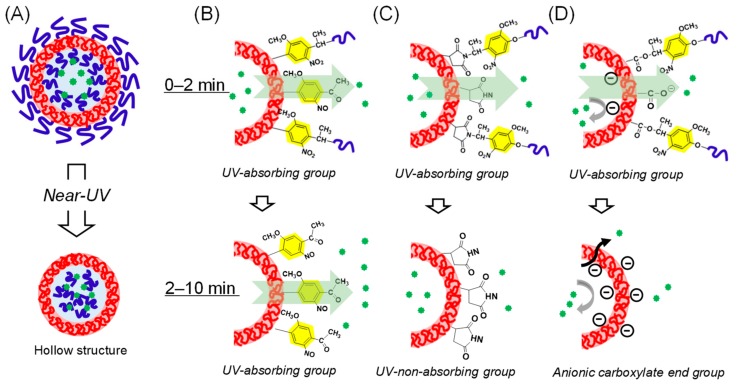
Summary and hypotheses of the photoreleasing mechanism from photoresponsive polymersomes. (**A**) All of the polymersomes retained their hollow structures even after sufficient irradiation. (**B–D**) Expected mechanisms of fluorescein photorelease from the polymersomes depending on photolysis residual groups and hydrophobic polymer species during initial 0–2 min (upper images) and subsequent 2–10 min (lower images). (**B**) Polymersomes **1a**–**c** and **2**, with the 2-nitrosoacetophenone end group, which continue absorbing near-UV light and exhibit prolonged light-induced payload release. (**C**) Polymersome **3**, with the non-UV-absorbing succinimide end group, releases only during the UV-absorbing 2-nitrobenzyl group exists. (**D**) Polymersomes **4** and **5**, with the anionic carboxylate end group, exhibit different photorelease behaviors depending on hydrophobic polymer species; polymersome **4**, with robust crystalline PS hydrophobic shells, shows absolutely no photorelease because of the electrostatic repulsion between fluorescein and negatively charged polymersomes interfaces. In contrast, in polymersome **5**, which has more flexible amorphous PMCL hydrophobic shells, the payload overcomes the electrostatic repulsion, and leads to the fastest photo-induced payload release within initial 2 min, followed by slight prolonged but slow payload release.

The summary and hypotheses of payload release from the tested polymersomes are shown in Figure 8A–D and Table 2, respectively. Although all of our polymersomes maintained their hollow structures after completion of the photolysis reaction (Figure 8A), we observed diverse payload releasing behaviors depending on the species of amphiphilic diblock copolymers (Figure 6). Polymersomes **1a**–**c** and **2** showed sustained payload release even after the completion of the photolysis of the 2-nitrobenzyl group, whereas it was completely terminated in the case of **3** at 2 min (Table 2, lower row). It should be noted that for **1b** and **3**, the hydrophilic PEG and hydrophobic PS segments were swapped, and the only difference between the polymersomes was that the residual photoproduct stayed on the hydrophobic shell either as 2-nitrosoacetophenone or as succinimide. Considering the fact that the 2-nitosoacetophenone moiety also absorbs near-UV light and that there was no spontaneous release by the cessation of photoirradiation at 2 min (Figure 6B, □), the absorption of near-UV light is critical for payload release and somehow leads to changes in the permeability of the hydrophobic shell (Figure 8B). The reciprocal relationship between the thickness of the hydrophobic PS shells and the rate of payload release (Table 2, **1a**–**c**), as determined from the SAXS and fluorescence dequenching experiments, respectively, are also a strong evidence for this hypothesis. On the other hand, **3** exhibited the photo-induced payload release only during 0–2 min, when 2-nitrobenzyl group remained within the hydrophobic shells (Figure 8C). In addition, the effect of the charged photolysis residues on the photoreleasing properties can be clearly seen by comparing polymersomes **3** and **4**, as each produces neutral succinimide and anionic carboxylate, respectively. The absolute lack of photorelease from **4** (Table 2) can be attributed to the electrostatic repulsion of the carboxylate photoproduct and the anionic fluorescein molecule (Figure 8D). Additionally, photoreleasing properties were also dependent on the type of the hydrophobic segments (Figure 8D). Although the permeation of the anionic payload though the hydrophobic PS shells was completely abolished in the polymersome **4** (*vide supra*), polymersome **5** with the PMCL block exhibited the fastest payload release within the initial 2 min (Table 2, 5, upper row). Moreover, this polymersome released the payload albeit only slightly even after the complete removal of the near-UV-absorbing 2-nitrobenzyl or 2-nitrosobenzyl functionalities from the hydrophobic shells (Table 2, 5, lower row). Considering the fact that this prolonged but slow release was not mediated by spontaneous leakage (Figure 6C, ◊), it is reasonable to conclude that the PMCL shells, with low glass transition temperature Tg (∼−60 °C) [35], are less resistant to the payload permeation than the robust PS barriers (Tg = 100 °C), thereby the weak energy transmitted through the light scattering by the nanoparticles can make the PMCL shells slightly permeable against the hydrophilic fluorophore. Regarding the structures of the polymersomes after photoirradiation, Burdick and coworkers demonstrated the maintenance of hollow structures and thickening of hydrophobic shells in the case of photocleavable polymersomes based on the PCL-PEG diblock bearing photocleavable 2-nitrobenzyl alanine at the hydrophobic/hydrophilic interface. Our results agreed with this previous work in terms of the maintenance of the hollow structures. However, further comprehensive comparison of the structural and kinetic properties is meaningless considering the essentially different photocleavage mechanisms between 2-nitrobenzyl amide (Burdick et al.) and 2-nitrobenyzlimide/2-nitrobenzyl ester (this work). In contrast, the chemical structure of the polymersomes used in this study was much closer to the polymersomes described by Meier and coworkers [22,23]. More specifically, in polymersome **5**, the hydrophobic block species (PMCL) and the photochemically produced end group at the polymersome surfaces (COOH) were the same. Nevertheless, photoirradiation led to completely different structures; the polymersomes synthesized by Meier and co-workers decomposed into smaller micelles, whereas ours retained their hollow spherical structures even after prolonged photoirradiation (Figure 7F). Therefore, even though the previous study attributed the driving force for polymersome decomposition to either the anionic negative charge generated at the polymersome surface or the amorphous nature of PMCL, this speculation does not seem to be applicable to our system (Figure 8D). There are still differences between the two studies, in terms of the molecular weight of each block (9500 vs. 6000) and hydrophilic block (PAAc vs. PEG). Therefore, further systematic syntheses and analyses may help elucidate the essential factors for the decomposition of the *linker*-type photoresponsive polymersomes. In this sense, our heterobifunctional photocleavable linkers (NBIL and NBEL) can serve as powerful tools for further investigations.

## 4. Conclusions

Here, we studied the structure and photo-release characteristics of a series of *linker*-type photoresponsive polymersomes with different polymer species and varying molecular weight of the hydrophobic block, as well as the residual groups of the polymersome surface after photocleavage. The common feature of these polymersomes was that they maintained their hollow structures even after the photolysis of the 2-nitrobenzyl group was complete. Remarkably, this was observed even for polymersomes **4** and **5**, for which the photolysis reaction produced a carboxy group at the surface of the hydrophobic shells, indicating that the mere introduction of carboxy group at the interfaces of polymersomes was not sufficient to decompose the overall structures of *linker*-type photoresponsive polymersomes. Interestingly, in the case of polymersomes **1a**–**c** and **2** with the residual 2-nitrosoacetophenone group, sustained photo-induced payload release was observed even after the completion of photolysis via unknown mechanisms. These results demonstrated the dependence of the payload release processes of the photoresponsive polymersomes on the chemical structures of the hydrophobic segments as well as the nature of photolysis residual groups. Such information will be useful for further developments of photoresponsive polymersomes with efficient drug releasing properties and good stability.

## Figures and Tables

**Table 1:** Characterization of photocleavable diblock copolymers.

	M_n_^a^	M_w_/M_n_^b^	Hydrophobic Polymer (M_n_) ^a^	PEG(wt %) ^a^
**1a**	5700	1.05	4100	13.2
**1b**	7600	1.07	6200	9.9
**1c**	9300	1.05	8000	8.0
**2**	7700	1.05	6300	9.7
**3**	8000	1.05	6500	9.4
**4**	7600	1.03	6200	9.9
**5**	8000	1.08	6600	9.4

^a^ Determined by ^1^H NMR. ^b^ Determined by gel permeation chromatography (GPC) using polystyrene standards.

**Table 2:** Averaged payload photorelease rates during initial 0–2 min (upper row) and subsequent 2–10 min (lower row).

	% of Fluorescein Released/Second						
	1a	1b	1c	2	3	4	5
0–2 min	0.22	0.16	0.059	0.16	0.11	< 0	0.26
2–10 min	0.11	0.070	0.062	0.080	< 0	< 0	0.030

## Supplementary Materials

The following are available online at https://www.mdpi.com/2073-4360/11/8/1254/s1, Figure S1. Synthetic routes of (A) NBEL, (B) thiol-terminated PMCL, and (C) photocleavable diblock copolymers **4** and **5**. Figure S2. GPC charts of photocleavable diblock copolymer (A) **4** and (B) **5** with RI detection. Figure S3. AF4 fractograms of polymersomes (A) **2**, (B) **3**, and (C) **5**. Figure S4. AF4 fractograms of (A) **1b**, (B) **2**, (C) **3**, and (D) **5** at a low flow rate (0.2 mL min^−1^). Figure S5. Structural characterization of the as-prepared polymersomes. Rg/Rh plots of (A) **2**, (B) **3**, and (C) **5**. Figure S6. ^1^H NMR of **1b** in CDCl3 (A) before irradiation, and after irradiation for (B) 2 min and (C) 10 min. Figure S7. Changes in the UV spectra of photocleavable diblock copolymer (A) **1b** and (B) **4** upon photoirradiation in THF. The polymer solution (0.1 mM) was irradiated with near-UV light (λ > 320 nm). Photolysis of the 2-nitrobenzyl ester was complete in 120 s, similar to the case of 2-nitrobenzylimide, because there were no further changes in the UV spectrum. Figure S8. Structural changes of polymersomes in response to photoirradiation. (A-C) DLS results of polymersomes (A) **3** and (B) **4**, irradiated for 0 (■), 2 (gray, ♦), and 15 min (∆). Figure S9. AF4 fractograms of polymersome (A) **3** and (B) **5** after irradiation for 15 min. Table S1. Results of the payload releasing behaviors of entrapped fluorescein molecules from the polymersomes **1b** (NBIL type) and **4** (NBEL type) in dark without photoirradiation.
