# Supplementary material for: Preparation of a Series of Photoresponsive Polymersomes Bearing Photocleavable a 2-nitrobenzyl Group at the Hydrophobic/Hydrophilic Interfaces and Their Payload Releasing Behaviors
Source: Polymers (Basel). 2019 Jul 29;11(8):1254. doi: 10.3390/polym11081254 (PMC6724059; doi:10.3390/polym11081254)
Supplement: Supplementary file 1 [file polymers-11-01254-s001.pdf]

Supplementary information for

Preparation of a series of photoresponsive polymersomes bearing photocleavable a 2-nitrobenzyl group at the hydrophobic/hydro-philic interfaces and their payload releasing behaviors

Shota Yamamoto [1,2], Takafumi Yamada [2], Genki Kubo [3], Kazuo Sakurai [3], Kazuo Yamaguchi [2,*] and Jun Nakanishi [1,4,*]

1 International Center for Materials Nanoarchitectonics (WPI-MANA), National Institute for Materials Science (NIMS), 1-1 Namiki, Tsukuba, Ibaraki 305-0044, Japan, E-mail: NAKANISHI.Jun@nims.go.jp

2 Department of Chemistry, Kanagawa University, 2946 Tsuchiya, Hiratsuka, Kanagawa 259-1293, Japan, E-mail: kazu@kanagawa-u.ac.jp

3 Department of Chemistry and Biochemistry, The University of Kitakyushu, 1-1 Hibikino, Wakamatsu-ku, Kitakyushu, Fukuoka 808-0135, Japan

4 Graduate School of Advanced Science and Engineering, Waseda University, 3-4-1 Okubo, Shinjuku-ku, Tokyo 169-8555, Japan

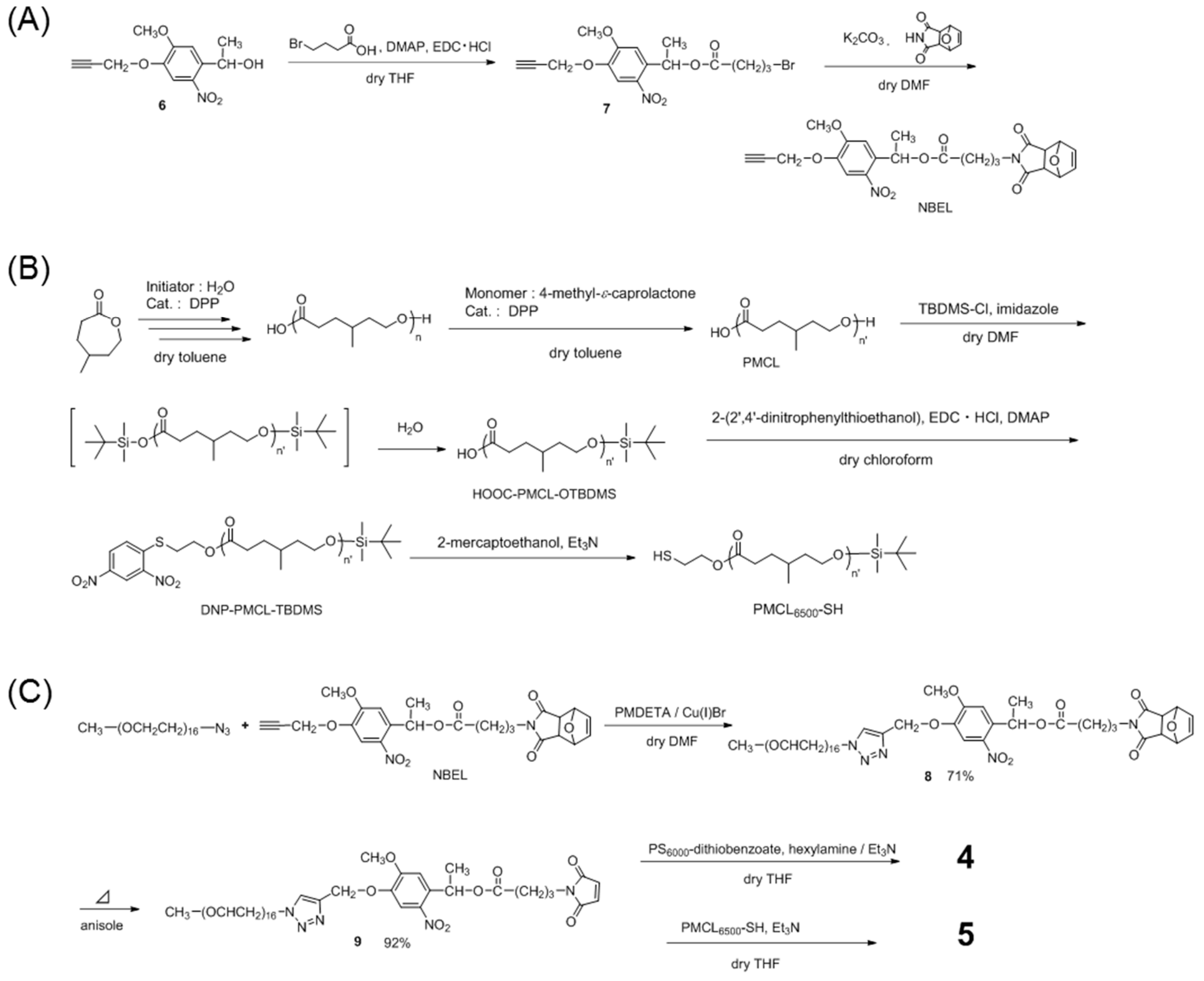

Figure S1. Synthetic routes of (A) NBEL, (B) thiol-terminated PMCL, and (C) photocleavable diblock copolymers **4** and **5**.

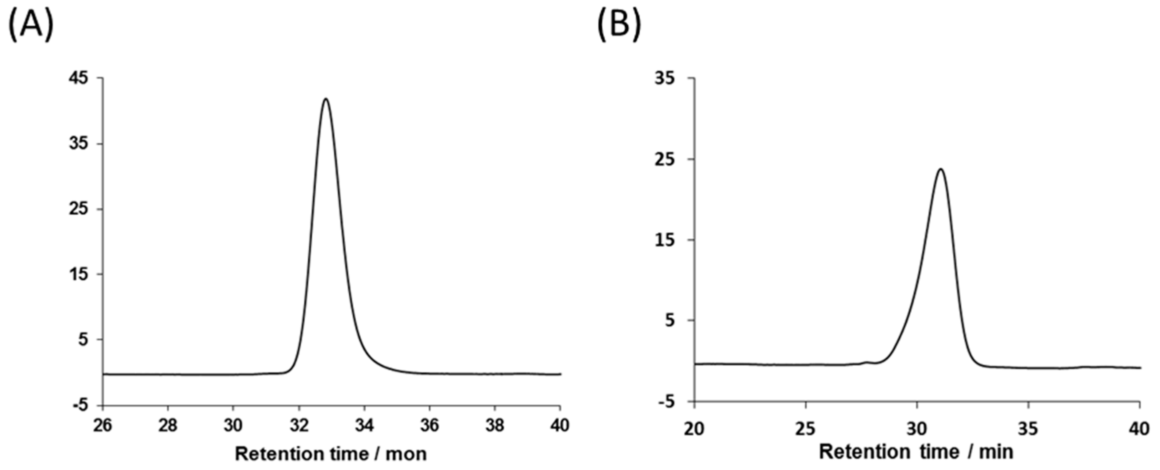

Figure S2. GPC charts of photocleavable diblock copolymer (A) **4** and (B) **5** with RI detection.

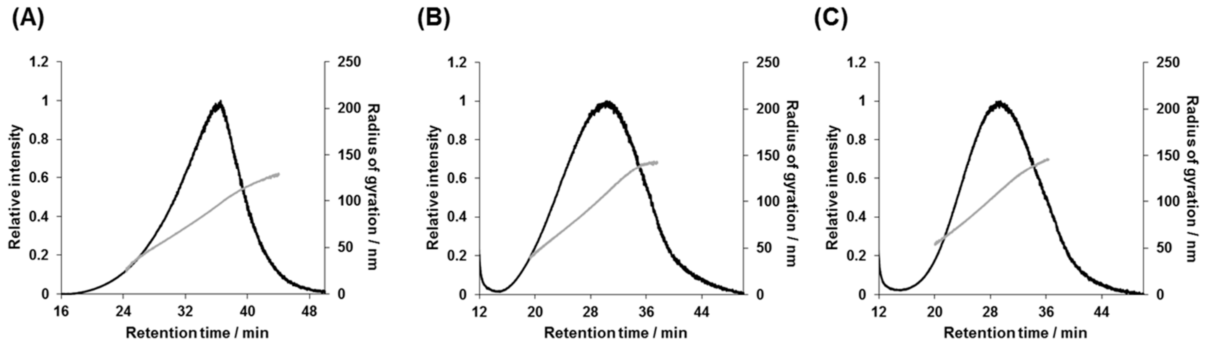

Figure S3. AF4 fractograms of polymersomes (A) **2**, (B) **3**, and (C) **5**.

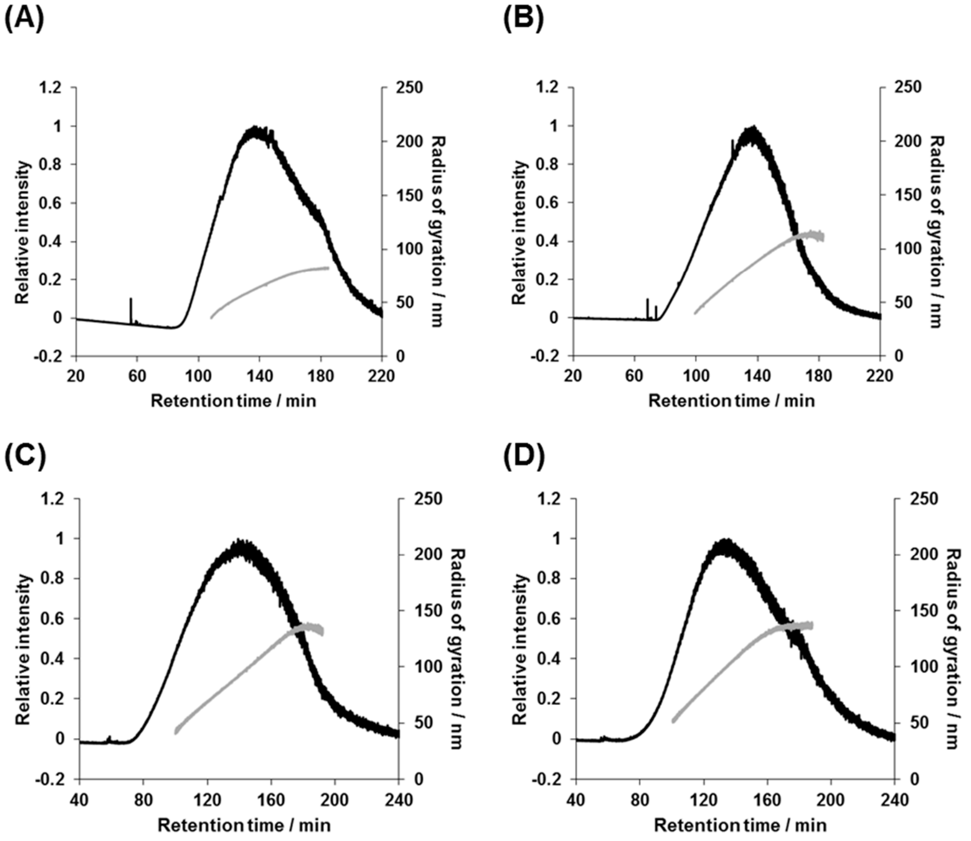

Figure S4. AF4 fractograms of (A) **1b**, (B) **2**, (C) **3**, and (D) **5** at a low flow rate (0.2 mL min^{-1}).

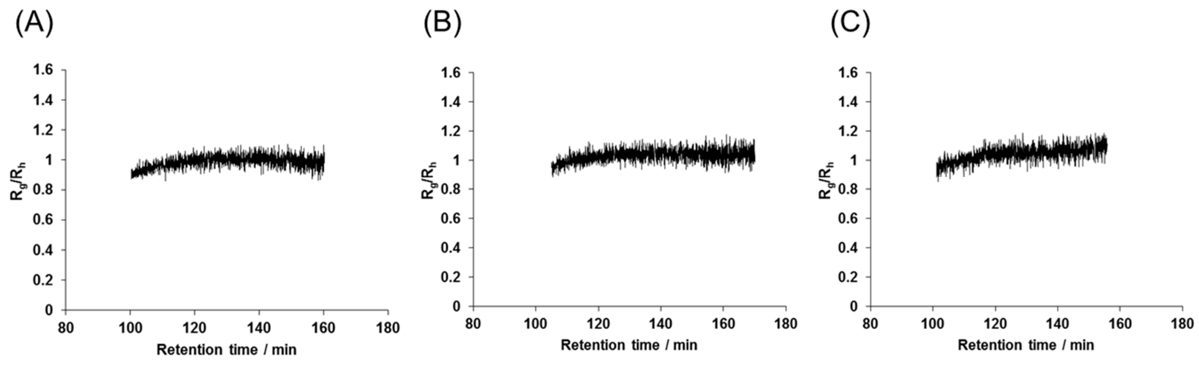

Figure S5. Structural characterization of the as-prepared polymersomes. R_g/R_h plots of (A) **2**, (B) **3**, and (C) **5**.

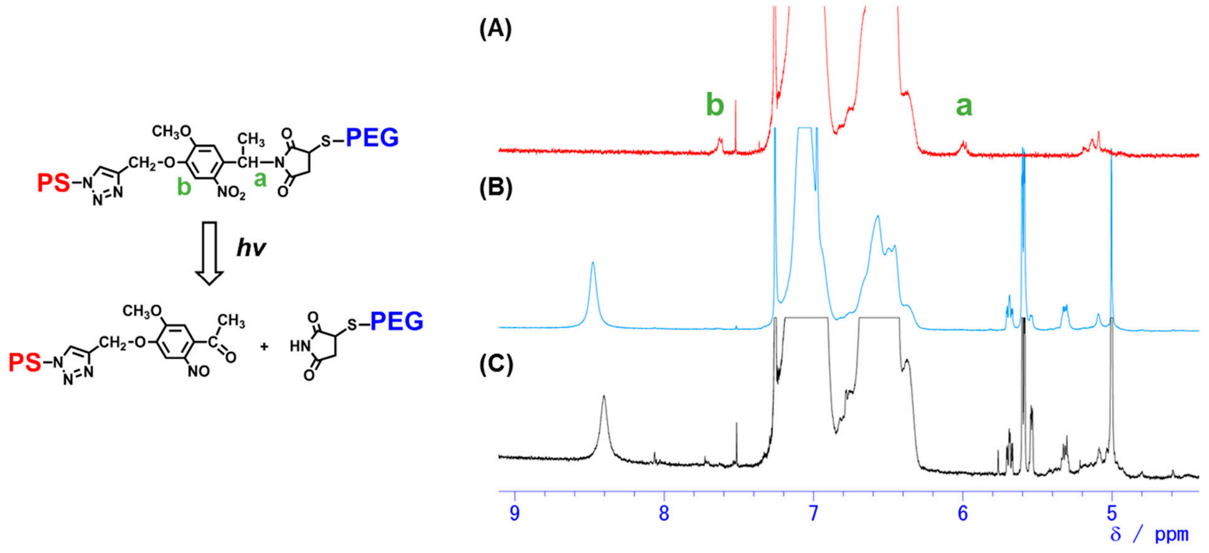

Figure S6. ^{1}H NMR of **1b** in $CDCl_3$ (A) before irradiation, and after irradiation for (B) 2 min and (C) 10 min.

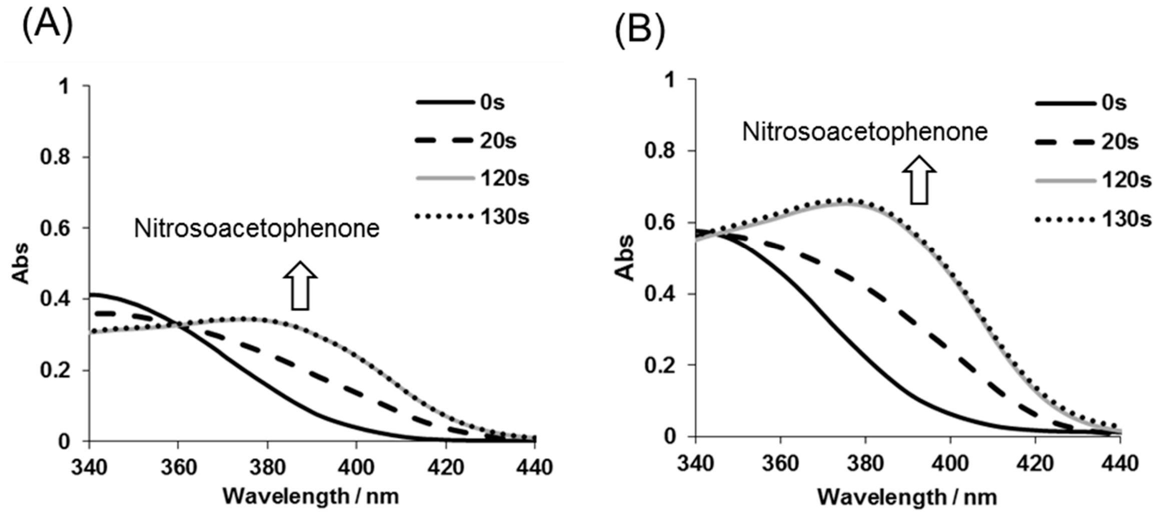

Figure S7. Changes in the UV spectra of photocleavable diblock copolymer (A) **1b** and (B) **4** upon photoirradiation in THF. The polymer solution (0.1 mM) was irradiated with near-UV light (λ > 320 nm). Photolysis of the 2-nitrobenzyl ester was complete in 120 s, similar to the case of 2-nitrobenzylimide, because there were no further changes in the UV spectrum.

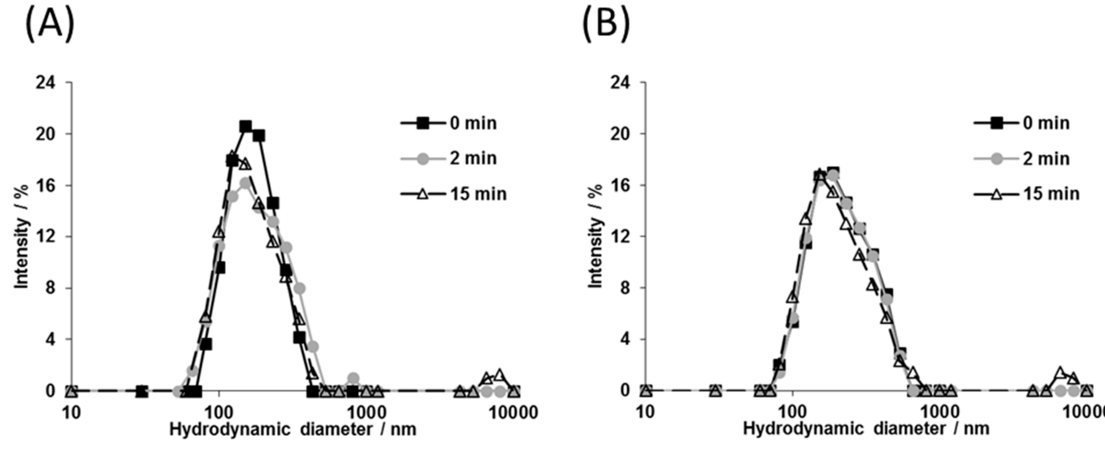

Figure S8. Structural changes of polymersomes in response to photoirradiation. (A-C) DLS results of polymersomes (A) **3** and (B) **4**, irradiated for 0 (■), 2 (gray, ♦), and 15 min (Δ).

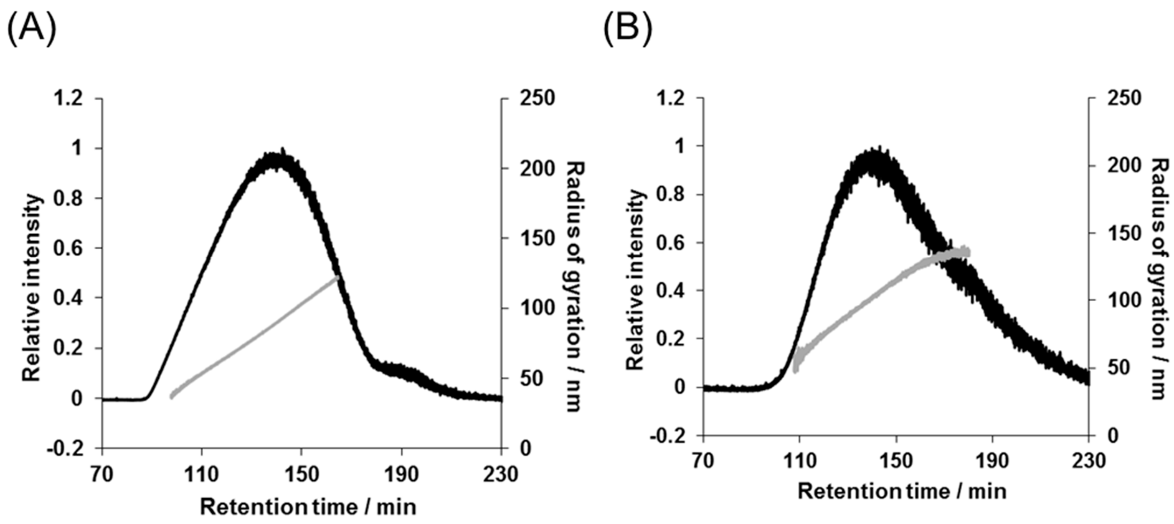

Figure S9. AF4 fractograms of polymersome (A) **3** and (B) **5** after irradiation for 15 min.

Table S1. Results of the payload releasing behaviors of entrapped fluorescein molecules from the polymersomes **1b** (NBIL type) and **4** (NBEL type) in dark without photoirradiation.

	% of fluorescein released	
	**1b**	**4**
5 min	1.55	1.29
10 min	1.12	1.69
